# Revisiting Reaction Mechanism of Regioselective Disulfide‐Catalyzed Photocatalytic Aerobic Oxidative Cleavage of 1‐Arylbutadienes: A Computational Study

**DOI:** 10.1002/cphc.202401004

**Published:** 2025-04-14

**Authors:** Meryem Fıstıkçı, Ferruh Lafzi, Selçuk Eşsiz

**Affiliations:** ^1^ Department of Medical Services and Techniques Vocational School of Health Services Hakkari University Hakkari 30000 Türkiye; ^2^ Department of Chemistry Faculty of Sciences Atatürk University Erzurum 25240 Türkiye

**Keywords:** aerobic oxidative cleavage, arylbutadienes, density functional theory, diaryl disulfides, DLPNO‐CCSD(T)

## Abstract

A computational study of the regioselective aerobic oxidative cleavage of 1‐arylbutadienes is carried out employing density functional theory and high‐level coupled‐cluster methods, such as coupled‐cluster singles and doubles with perturbative triples [CCSD(T)]. The results demonstrate that the reaction proceeds either via the intramolecular reduction or dimerization of peroxyl radical. These findings are in contrast to a previously proposed mechanism that progresses via formation of the dioxetane ring. The computations further indicate that the homolysis of S—S bond of diaryl disulfide derivatives cannot be achieved by irradiation with direct visible light under the reaction conditions due to the high bond dissociation energy.

## Graphical abstract

This study demonstrates that the regioselective aryldisulfide‐catalyzed photocatalytic aerobic oxidative cleavage of 1‐arylbutadienes progresses through reduction or dimerization of peroxyl radical contrary to the formation of dioxetane suffering from the ring strain.

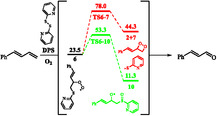

## 1 Introduction

**Scheme 1:**
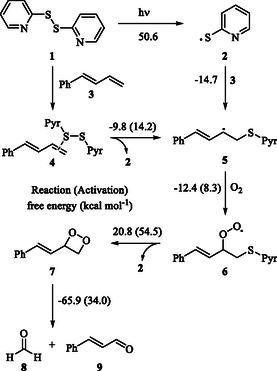
Reaction mechanism proposed by Fernandes and co‐workers, evaluated with computational methods. The given energy values (kcal mol^–^^1^) are derived from our study.

The aerobic oxidative cleavage of C—C double bonds, a key focus point for sustainable chemistry and an alternative to ozonolysis, is one of the most effective methods for the synthesis of carbonyl compounds. The C—C double bond cleavage of alkenes strategically provides new opportunities in the synthesis of carbonyl compounds, especially metal‐free methods. Over the years, many organocatalysts have been developed for the synthesis of carbonyl compounds by the aerobic oxidative cleavage of C—C double bonds.^[^ ^1^, ^2^, ^3^, ^4^, ^5^, ^6^, ^7^, ^8^, ^9^, ^10^, ^11^, ^12^ ^]^ In 2022, Fernandes and co‐workers^[^ ^12^ ^]^ developed a photocatalytic method for regioselective aerobic oxidative cleavage of 1‐arylbutadienes by using various diaryl disulfides (as photocatalyst) in the presence of visible light. With this method, one of the double bonds in 1‐arylbutadienes is selectively oxidized by moleculer oxygen, yielding cinnamaldehyde derivatives with numerous synthetic applications and biological activities.^[^ ^13^, ^14^, ^15^, ^16^, ^17^ ^]^ 2,2′‐Dipyridyl disulfide (5 mol%) was utilized as the photocatalyst in acetone at room temperature in the presence of white LED (9 W) light and oxygen atmosphere as the optimum conditions for the regioselective aerobic oxidative cleavage of 1‐arylbutadienes. In addition, selective control experiments showed that the photocatalytic aerobic oxidation of 1‐arylbutadienes proceeds via a radical chain process and the oxygen atom in cinnamaldehyde derivatives comes mostly from the molecular oxygen, the mechanism of which is outlined in **Scheme** **1**.

Preliminary mechanistic understandings^[^ ^1^, ^12^ ^]^ invoked initial formation of the 2‐pyridyl sulfide radical **(2)** generated by the homolytic cleavage of the disulfide bond of 2,2′‐dipyridyl disulfide **(1)** via visible light irradiation. Then, an attack of 2‐pyridyl sulfide radical **(2)** to the terminal carbon of the olefin of 1‐phenylbutadiene **(3)** yields intermediate **5** which is trapped by O_2_ to form the corresponding peroxyl radical **6**. Homolysis of the carbon‐sulfur bond to reform 2‐pyridyl sulfide radical **(2)** completes the catalytic and subsequent cyclization of the peroxyl radical generates dioxetane derivative **7**. Finally, ring‐opening reaction of dioxetane derivative **7** yields the final product, cinnamaldehyde **(9)** and formaldehyde **(8)** (Scheme 1).

Herein, we disclose a computational study employing density functional theory (DFT) and high‐level coupled‐cluster methods for the regioselective aerobic oxidative cleavage of 1‐arylbutadienes to cinnamaldehydes via an arylsulfide radical. Both the mechanism of Fernandes and co‐workers and an alternative more plausible mechanism suggested by us were taken into consideration.

## 2 Results and Discussion

**Figure 1:**
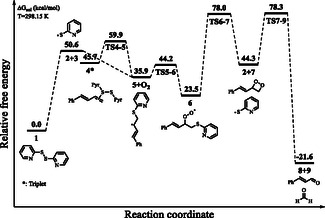
Relative free energy profile for the reaction mechanism shown in Scheme 1.

**Figure 2:**
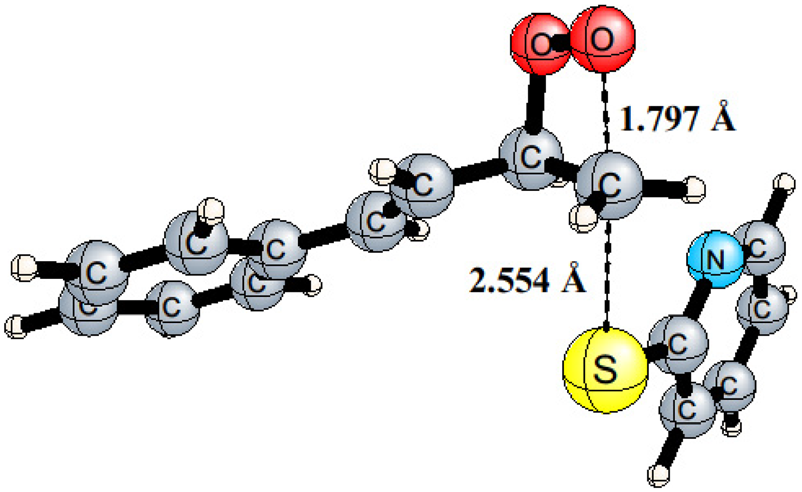
Computed structure of **TS6‐7** at the B3LYP‐D3/6‐311G(d,p)/SMD(acetone) level.

All computations were carried out using DFT at the B3LYP‐D3/6‐311G(d,p)/SMD (acetone) level, and single‐point energy calculations were performed with the DLPNO‐CCSD(T)/SMD (acetone) method employing the cc‐pVTZ basis set. Solvent effects were considered using the SMD model in acetone. Further computational details can be found in the Experimental Section and Supporting Information. Our results for the mechanism proposed by Fernandes and co‐workers are reported in **Figure** **1**. For the initiation of the radical cycle, the **1** **→** **2** conversion, the computed reaction free energy was found to be 50.6 kcal mol^−1^ (Figure 1). Additionally, no transition state (TS) was found for this step. The reaction progresses via a barrierless pathway for the attack of 2‐pyridyl sulfide radical **(2)** onto the terminal olefinic carbon of 1‐phenylbutadiene **(3)**, **3 →** **5**, while the reaction free energy is −14.7 kcal mol^−1^. For the formation of peroxyl radical **6** via trapping of O_2_ by the radical **5**, **5** **→** **6**, the reaction and activation free energies are −12.4 and 8.3 kcal mol^−1^, respectively. For the formation of dioxetane derivative **7** via ring‐closure reaction, **6** **→** **7**, the reaction and activation free energies are 20.8 and 54.5 kcal mol^−1^, respectively. For the formation of cinnamaldehyde **(9)** via the retrocycloaddition reaction of the dioxetane derivative **7**, **7** **→** **9**, the reaction and activation free energies are −65.9 and 34.0 kcal mol^−1^, respectively (Figure 1). The optimized geometry of the key TS **TS6–7** with selected interatomic distances is shown in **Figure** **2**.

While our study primarily focuses on the reaction pathway following the formation of intermediate **6**, alternative initiation pathways for the homolysis of the S—S bond under visible light conditions remain an open question, except for disulfide–olefin complex. Future studies may explore the role of potential photosensitizers or charge‐transfer complexes in facilitating this bond dissociation. Our computed reaction free energy for the formation of radical **2** is 50.6 kcal mol^−1^. This value is very high for the reported reaction conditions. More importantly, the homolysis of S—S bond of organic disulfides cannot be achieved simply via irradiation with visible light as the homolysis occurs via irradiation with UV light.^[^ ^10^, ^18^ ^]^ Additionally, our computations show that the reaction activation free energy for the formation of dioxetane derivative **7** from the peroxyl radical **6** is also very high (54.5 kcal mol^−1^) (Figure 1). However, the reaction activation free energy for the reverse step, **7** **→** **6**, is 33.7 kcal mol^−1^, which is significantly lower than that for the forward step. The barrier for the formation of dioxetane derivative **7** is relatively high under the reaction conditions, which means that the reaction does not appear to proceed via the formation of dioxetane derivative **7**.^[^ ^19^, ^20^ ^]^ Furthermore, the homolytic S—S bond dissociation of olefin‐activated disulfide **4** under visible light and a subsequent thiyl addition to the C—C double bond yields intermediate **5**.^[^ ^10^ ^]^ Detailed computations for this complex were previously carried out by Wang and co‐workers.^[^ ^10^ ^]^ For the **4** **→** **5** conversion, the reaction and activation free energies are −9.8 and 14.2 kcal mol^−1^, respectively (Figure 1).

**Scheme 2:**
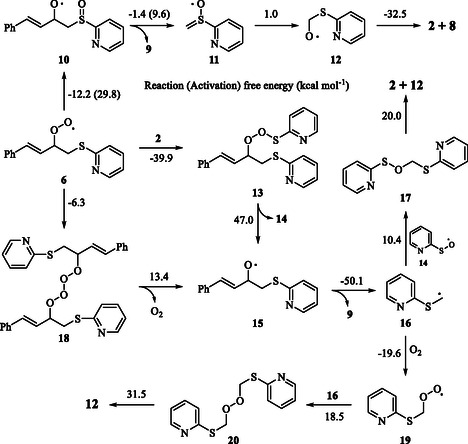
Proposed reaction mechanism for the regioselective aerobic oxidative cleavage of 1‐phenylbutadiene. The given energy values are (kcal mol^−^^1^).

**Figure 3:**
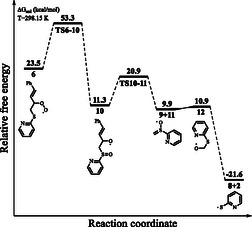
Relative free energy profile for the reaction mechanism of intramolecular reduction shown in Scheme 2.

According to our proposed mechanisms in **Scheme** **2**, the reaction can progress from intermediates **10**, **13**, or **18** via three different pathways. The intramolecular reduction of peroxyl radical **6** results in the formation of intermediate **10**.^[^ ^21^ ^]^ For the reduction of peroxyl radical **6**, **6 →** **10**, the computed reaction and activation free energies are −12.2 and 29.8 kcal mol^−1^, respectively. The elimination of cinnamaldehyde **(9)** from intermediate **10** yields intermediate **11**. For the **10** **→** **11** conversion, the reaction and activation free energies are −1.4 and 9.6 kcal mol^−1^, respectively. The rearrangement of intermediate **11** furnishes intermediate **12**. For this rearrangement, **11** **→** **12**, the computed reaction free energy is 1.0 kcal mol^−1^. The ejection of 2‐pyridyl sulfide radical **(2)** from intermediate **12** yields formaldehyde **(8)**. For the **12 →** **8** conversion, the reaction free energy is −32.5 kcal mol^−1^ (**Figure** **3**).

**Figure 4:**
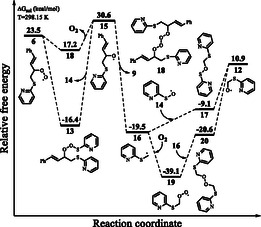
Relative free energy profile for the alternative reaction mechanism shown in Scheme 2.

As an alternative to intramolecular reduction for intermediate **6**, quenching and dimerization reactions are also possible as shown in Scheme 2. The trapping of 2‐pyridyl sulfide radical **(2)** by the peroxyl radical **6** yields intermediate **13**. For the formation of intermediate **13**, **6 + 2** **→** **13**, the computed reaction free energy is −39.9 kcal mol^−1^, while the reaction proceeds via a barrierless pathway. The elimination of intermediate **14** from intermediate **13** yields alkoxy radical **15**. For the **13 →** **15** conversion, the reaction free energy is 47.0 kcal mol^−1^. An alternative pathway for the formation of alkoxy radical **15** is also the ejection of molecular oxygen from intermediate **18** formed with self‐coupling of peroxyl radical **6**. These reactions proceed via a barrierless pathway and for the **6 →** **21 →** **20 + O**_**2**_ conversions, the computed reaction free energies are −6.3 and 13.4 kcal mol^−1^, respectively. The homolysis of intermediate **16** from intermediate **15** gives the main product, cinnamaldehyde **(9)**. For the formation of cinnamaldehyde **(9)**, the reaction free energy is −50.1 kcal mol^−1^. Finally, the radical–radical coupling of intermediate **16** and intermediate **14** formed by leaving intermediate **13** produces sulfenic ester derivative **17**.^[^ ^22^, ^23^, ^24^ ^]^ For the formation of sulfenic ester **17**, the reaction free energy is 10.4 kcal mol^−1^. The elimination of 2‐pyridyl sulfide radical **(2)** produces intermediate **12**. For the **17 →** **12** conversion, the computed reaction free energy is 20.0 kcal mol^−1^. Alternative route for intermediate **16** formed from intermediate **18** is the trapping of molecular oxygen. Peroxide derivative **20** is yielded via the radical coupling reaction following the trapping of molecular oxygen by intermediate **16**. For the **16 →** **19 →** **20** conversions, the computed reaction free energies are −19.6 and 18.5 kcal mol^−1^, respectively. The homolysis of peroxide **20** results in the formation of intermediate **12**. For the homolysis reaction, **20 →** **12**, the computed reaction free energy is 31.5 kcal mol^−1^ (**Figure** **4**).

## 3 Conclusion

In this study, the regioselective aerobic oxidative cleavage of 1‐arylbutadienes has been investigated with high‐level coupled‐cluster methods, such as DLPNO‐CCSD(T), along with the cc‐pVTZ basis set. Our results demonstrate that regioselective aryldisulfide‐catalyzed photocatalytic aerobic oxidative cleavage of 1‐arylbutadienes progresses through reduction or dimerization of peroxyl radical contrary to the formation of dioxetane suffering from the ring strain. Our results indicate the existence of a charge‐transfer complex between the disulfide and olefin during the unconventional homolysis of the S—S bond in diaryl disulfide derivatives under visible light conditions. Although our computations suggest that direct homolysis of the S—S bond under visible light conditions is unlikely, a detailed investigation of alternative initiation pathways, such as photosensitization or charge‐transfer activation, would be beneficial for a comprehensive understanding of the process. These findings are in agreement with the experimental results for the regioselective aerobic oxidative cleavage of 1‐arylbutadienes and homolysis of the S—S bond of diaryl disulfides.

## 4 Computational Methods

Geometry optimizations and harmonic vibrational frequency computations were performed using DFT at the B3LYP‐D3 level, employing the Pople‐type polarized triple‐ζ split‐valence basis set, 6‐311G(d,p).^[^ ^25^, ^26^, ^27^, ^28^, ^29^, ^30^, ^31^ ^]^ Single‐point energy calculations were conducted at the optimized geometries using the domain‐based local pair natural orbitals coupled‐cluster method [DLPNO‐CCSD(T)] with the cc‐pVTZ basis set.^[^ ^32^, ^33^, ^34^, ^35^ ^]^ Solvation effects were modeled using the solvation model based on density (SMD) in acetone (*ε* = 20.493) for all computational methods.^[^ ^36^, ^37^ ^]^ The TSs were confirmed to connect to reactants and products by intrinsicreaction coordinate computations.^[^ ^38^, ^39^ ^]^

Additionally, single‐point energy corrections were computed at the SMD/wB97XD‐def2TZVP and SMD/M062X‐D3‐6‐311++G(d,p) levels (see Supporting Information). The TS structures are denoted as TSA‐B throughout the article. Optimized geometries of the TSs with selected interatomic distances, Cartesian coordinates, and relative free energy profiles are provided in the Supporting Information.

All DFT computations were performed using Gaussian 16,^[^ ^40^ ^]^ while DLPNO‐CCSD(T) calculations were carried out with ORCA 4.2.1.^[^ ^41^, ^42^ ^]^ Additionally, the open‐source software cheMVP.exe was used for 3D structure visualization.

## Conflict of Interest

The authors declare no conflict of interest.

## Supporting information

Supplementary Material

## Data Availability

The data that support the findings of this study are available in the supplementary material of this article.
