# Supplementary material for: Revisiting Reaction Mechanism of Regioselective Disulfide‐Catalyzed Photocatalytic Aerobic Oxidative Cleavage of 1‐Arylbutadienes: A Computational Study
Source: Chemphyschem. 2025 Apr 14;26(11):e202401004. doi: 10.1002/cphc.202401004 (PMC12132911; doi:10.1002/cphc.202401004)
Supplement: Supplementary file 1 — Supplementary Material [file CPHC-26-e202401004-s001.pdf]

Revisiting Reaction Mechanism of Regioselective Disulfide-catalyzed Photocatalytic Aerobic Oxidative Cleavage of 1-Arylbutadienes: A Computational Study

Meryem Fıstıkçı,*[a] Ferruh Lafzi,[b] Selçuk Eşsiz[a]

[a]*Department of Medical Services and Techniques, Vocational School of Health Services, Hakkari University, Hakkari, 30000, Türkiye*

[b]*Department of Chemistry, Faculty of Sciences, Atatürk University, 25240, Erzurum, Türkiye*

meryemfistikci@hakkari.edu.tr

Table of contents

1. The Optimized Geometries of the Transition States with Selected Interatomic Distances 3

Figure S1. Computed structure of **TS4-5** at the B3LYP/6-311G(d,p) level (im. freq.= 415.96*i* cm^{-1})........... 3

Figure S2. Computed structure of **TS5-6** at the B3LYP/6-311G(d,p) level (im. freq.= 187.38*i* cm^{-1})........... 3

Figure S3. Computed structure of **TS6-7** at the B3LYP/6-311G(d,p) level (im. freq.= 1602.96*i* cm^{-1})......... 3

Figure S4. Computed structure of **TS7-9** at the B3LYP/6-311G(d,p) level (im. freq.= 1269.81*i* cm^{-1})......... 4

Figure S5. Computed structure of **TS6-10** at the B3LYP/6-311G(d,p) level (im. freq.= 1007.13*i* cm^{-1})....... 4

Figure S6. Computed structure of **TS10-11** at the B3LYP/6-311G(d,p) level (im. freq.= 411.24*i* cm^{-1})....... 4

2. Relative Free Energy Profiles 5

Figure S7. Relative free energy profile (wB97XD-def2-TZVP//B3LYP-6-311G(d,p)) for the reaction mechanism shown in Scheme 1. 5

Figure S8. Relative free energy profile (wB97XD-def2-TZVP//B3LYP-6-311G(d,p)) for the reaction mechanism shown in Scheme 2. 6

Figure S9. Relative free energy profile (wB97XD-def2-TZVP//B3LYP-6-311G(d,p)) for the reaction mechanism shown in Scheme 2 7

Figure S10. Relative free energy profile (M06X-D3-6-311++G(d,p)//B3LYP-6-311G(d,p)) for the reaction mechanism shown in Scheme 1. 8

Figure S11. Relative free energy profile (M06X-D3-6-311++G(d,p)//B3LYP-6-311G(d,p)) for the reaction mechanism shown in Scheme 2. 9

Figure S12. Relative free energy profile (M06X-D3-6-311++G(d,p)//B3LYP-6-311G(d,p)) for the reaction mechanism shown in Scheme 2 10

3. Single Point Energy Computations 11

Table S1. Single point energies (**E**) for species using DLPNO-CCSD(T)/cc-PVTZ level in a.u. 11

Table S2. Single point energies (**E**) for species using wB97XD-def2-TZVP//B3LYP-6-311G(d,p) level in a.u. 12

Table S3. Single point energies (**E**) for species using M06X-D3-6-311++G(d,p)//B3LYP-6-311G(d,p) level in a.u. 13

4. Cartesian Coordinates for Optimized Structures 14

1. The Optimized Geometries of the Transition States with Selected Interatomic Distances

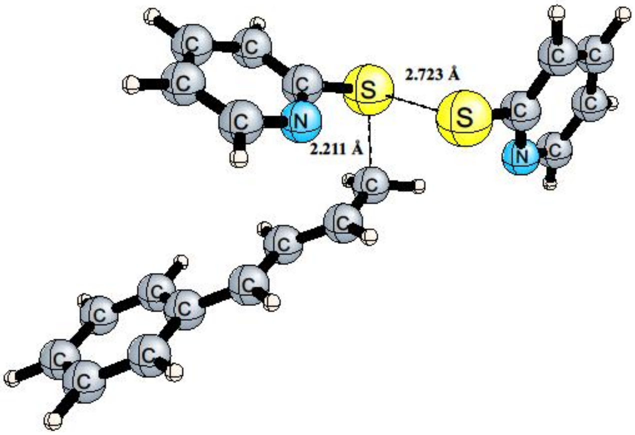

Figure S1. Computed structure of **TS4-5** at the B3LYP/6-311G(d,p) level (im. freq.= 415.96*i* cm^{-1}).

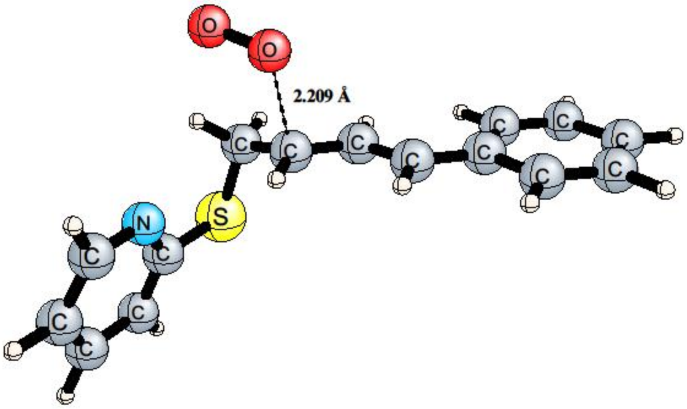

Figure S2. Computed structure of **TS5-6** at the B3LYP/6-311G(d,p) level (im. freq.= 187.38*i* cm^{-1}).

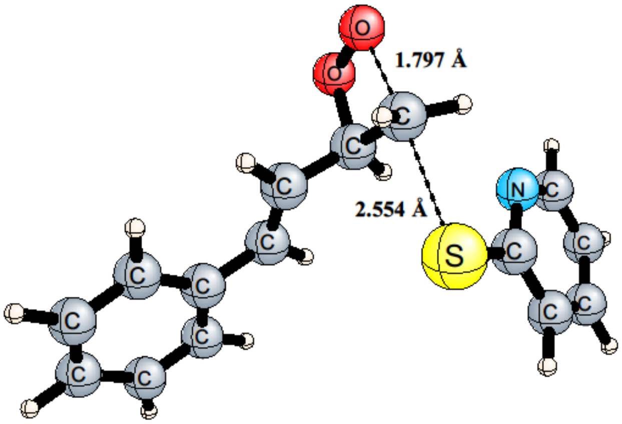

Figure S3. Computed structure of **TS6-7** at the B3LYP/6-311G(d,p) level (im. freq.= 1602.96*i* cm^{-1}).

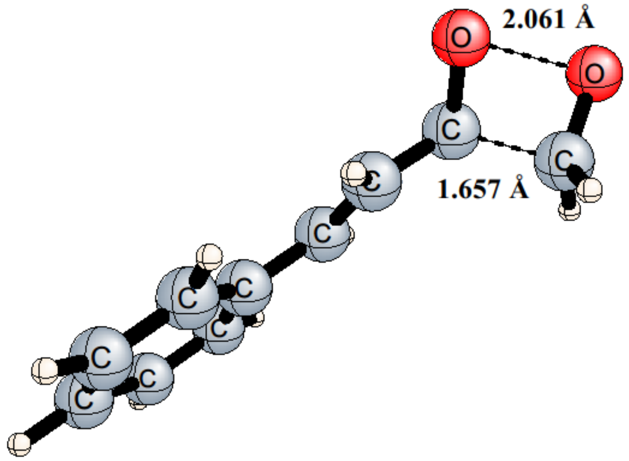

Figure S4. Computed structure of **TS7-9** at the B3LYP/6-311G(d,p) level (im. freq.= 1269.81*i* cm^{-1}).

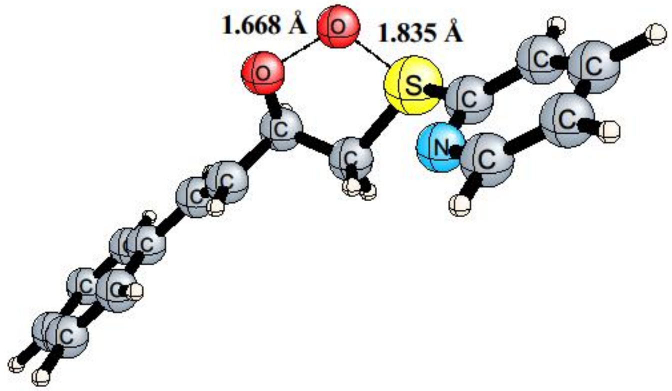

Figure S5. Computed structure of **TS6-10** at the B3LYP/6-311G(d,p) level (im. freq.= 1007.13*i* cm^{-1}).

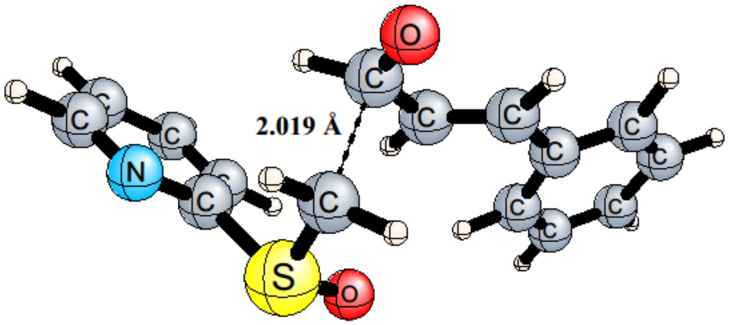

Figure S6. Computed structure of **TS10-11** at the B3LYP/6-311G(d,p) level (im. freq.= 411.24*i* cm^{-1}).

2. Relative Free Energy Profiles

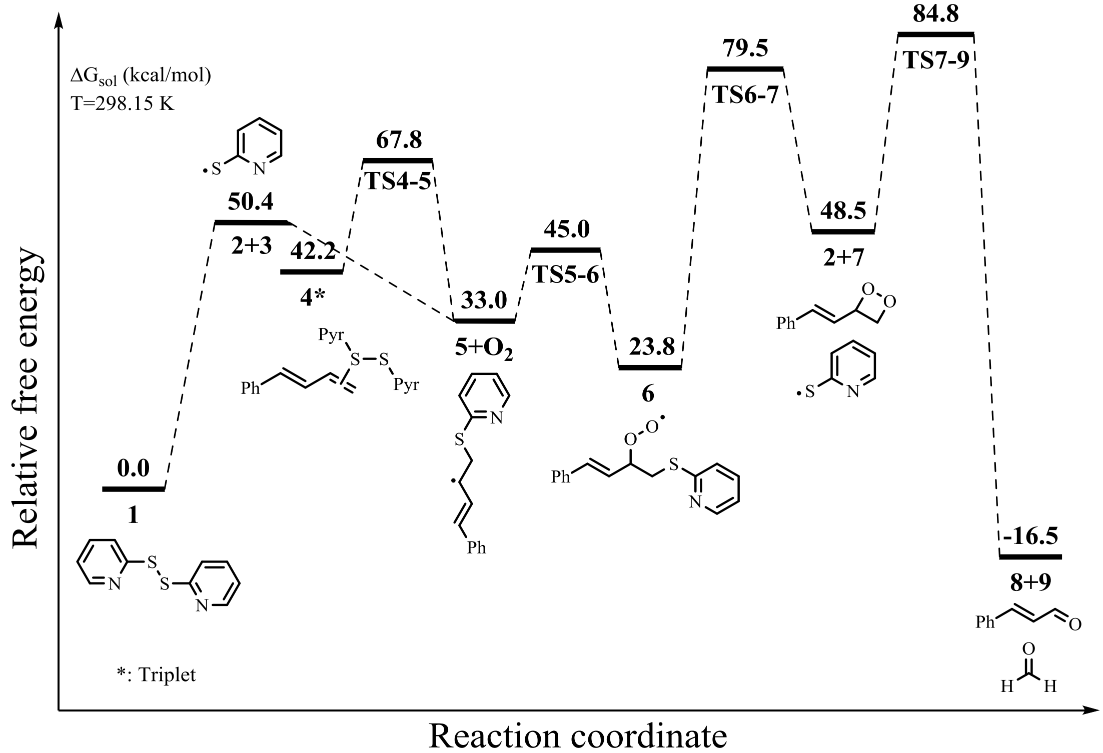

Figure S7. Relative free energy profile (wB97XD-def2-TZVP//B3LYP-6-311G(d,p)) for the reaction mechanism shown in Scheme 1.

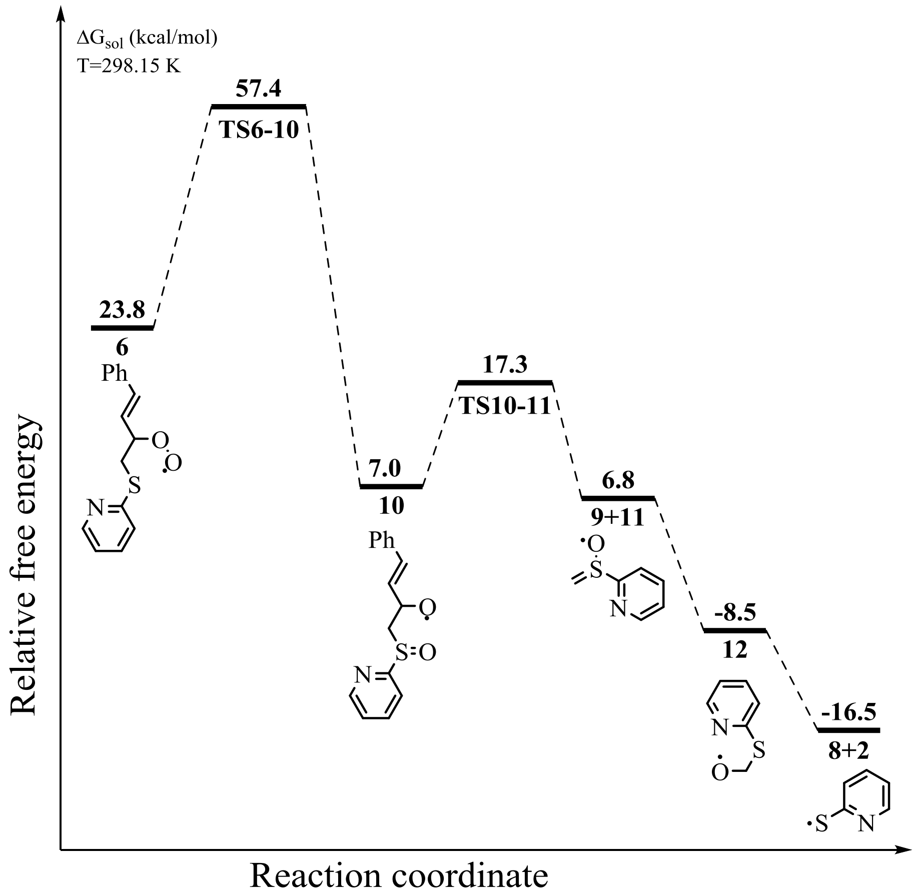

Figure S8. Relative free energy profile (wB97XD-def2-TZVP//B3LYP-6-311G(d,p)) for the reaction mechanism shown in Scheme 2.

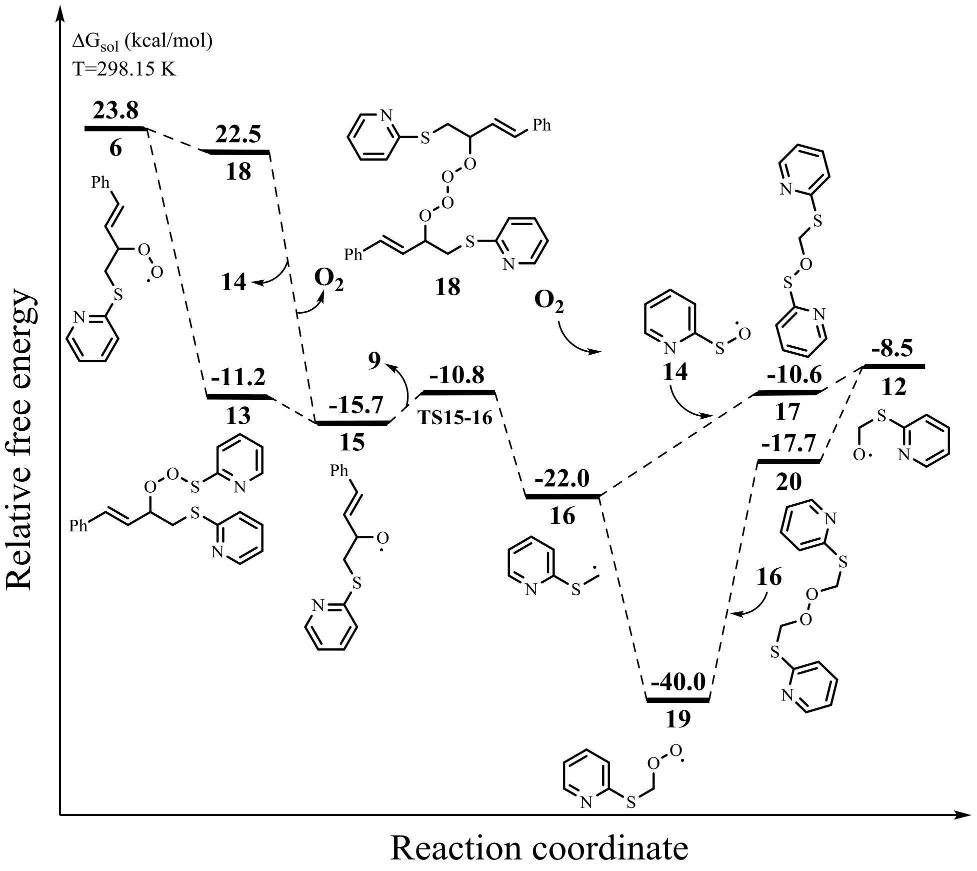

Figure S9. Relative free energy profile (wB97XD-def2-TZVP//B3LYP-6-311G(d,p)) for the reaction mechanism shown in Scheme 2

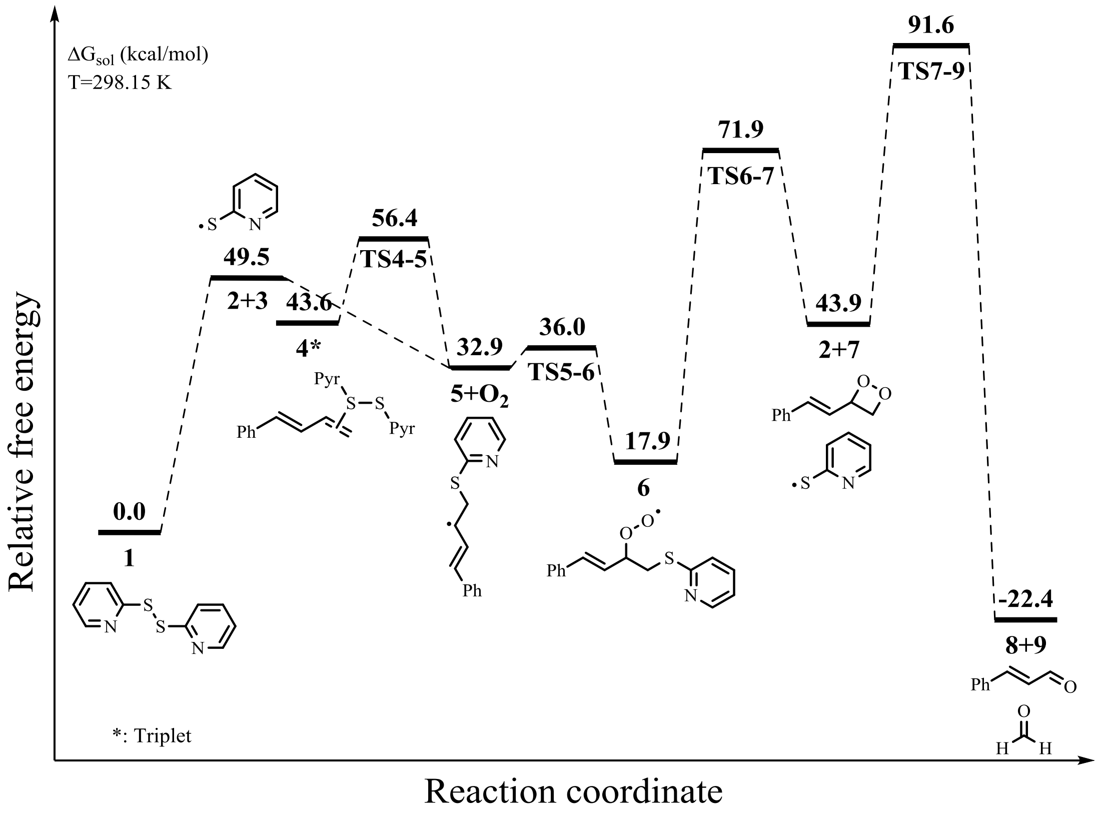

Figure S10. Relative free energy profile (M06X-D3-6-311++G(d,p)//B3LYP-6-311G(d,p)) for the reaction mechanism shown in Scheme 1.

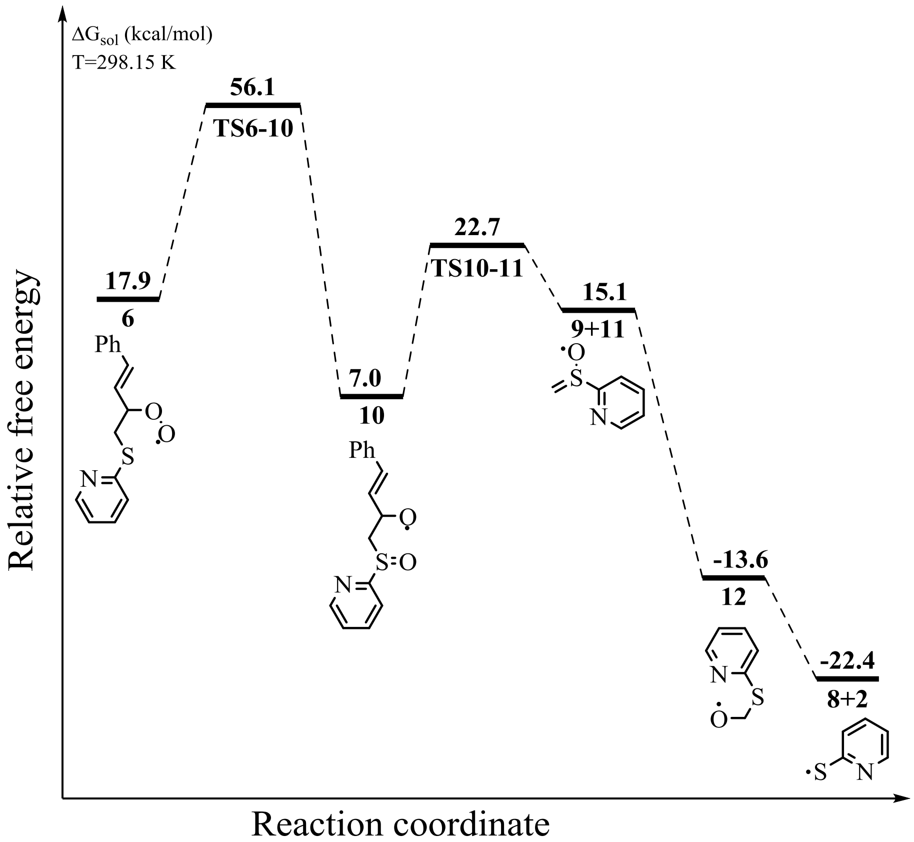

Figure S11. Relative free energy profile (M06X-D3-6-311++G(d,p)//B3LYP-6-311G(d,p)) for the reaction mechanism shown in Scheme 2.

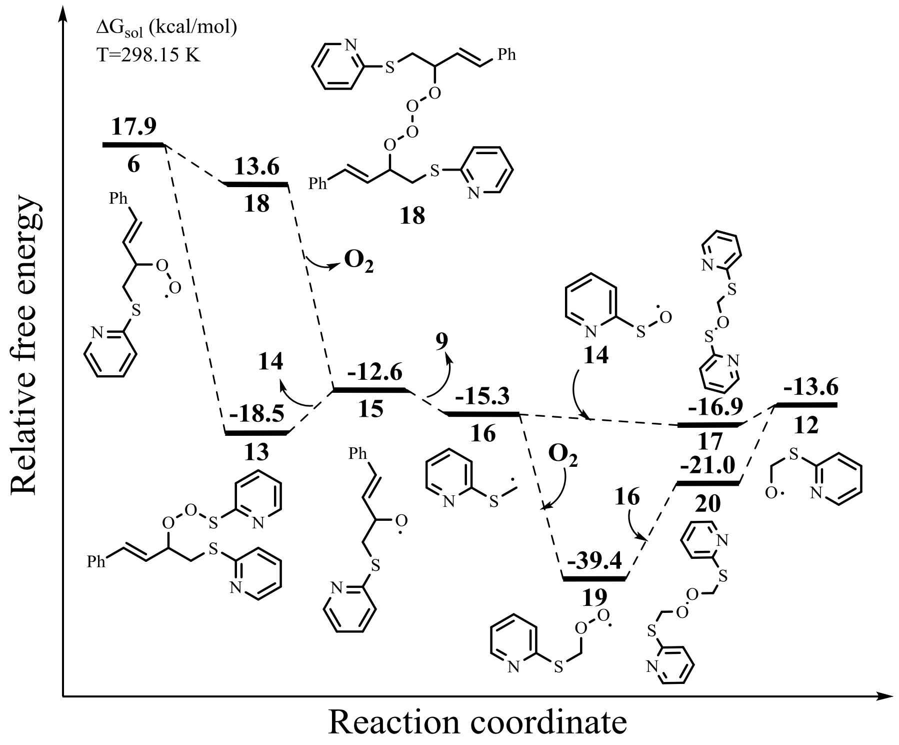

Figure S12. Relative free energy profile (M06X-D3-6-311++G(d,p)//B3LYP-6-311G(d,p)) for the reaction mechanism shown in Scheme 2

3. Single Point Energy Computations

Table S1. Single point energies (**E**) for species using DLPNO-CCSD(T)/cc-PVTZ level in a.u.

Molecule	E	TS	E
**oxygen**	-150.12842301	**TS4-5**	-1676.19007923
**1**	-1289.96123692	**TS5-6**	-1181.40460208
**2**	-644.93711391	**TS6-7**	-1181.35153170
**3**	-386.32065036	**TS7-9**	-536.40937342
**3***	-386.23756111	**TS6-10**	-1181.393479008
**4***	-1676.20897257	**TS10-11**	-1181.44414537
**5**	-1031.28763195		
**6**	-1181.44162352		
**7**	-536.46759076		
**8**	-114.33827146		
**9**	-422.22428484		
**10**	-1181.46093029		
**11**	-759.22815349		
**12**	-759.22834793		
**13**	-1826.45074224		
**14**	-720.05943693		
**15**	-1106.30670129		
**16**	-684.15304921		
**17**	-1404.28355396		
**17***	-1404.19915933		
**18**	-2362.91595099		
**19**	-834.32007119		
**20**	-1518.57709884		
**20***	-1518.44685645		

***Triplet**

Table S2. Single point energies (**E**) for species using wB97XD-def2-TZVP//B3LYP-6-311G(d,p) level in a.u.

**Molecule**	**E**	**TS**	**E**
**oxygen**	-150.34678440	**TS4-5**	-1678.76046180
**1**	-1291.80238290	**TS5-6**	-1183.28398760
**2**	-645.85787510	**TS6-7**	-1183.22963860
**3**	-387.06247550	**TS7-9**	-537.35881680
**3***	-386.98448370	**TS6-10**	-1183.26749800
**4***	-1678.79755710	**TS10-11**	-1183.33033640
**5**	-1032.95454770		
**6**	-1183.32170220		
**7**	-537.42057880		
**8**	-114.51650360		
**9**	-422.99765720		
**10**	-1183.34838140		
**11**	-760.34026780		
**12**	-760.36638600		
**13**	-1829.24369800		
**14**	-721.09315890		
**15**	-1108.14820150		
**16**	-685.15124930		
**17**	-1406.30662780		
**17***	-1406.22950080		
**18**	-2366.66040920		
**19**	-835.53403580		
**20**	-1520.77924370		
**20***	-1520.65291330		

***Triplet**

Table S3. Single point energies (**E**) for species using M06X-D3-6-311++G(d,p)//B3LYP-6-311G(d,p) level in a.u.

**Molecule**	**E**	**TS**	**E**
**oxygen**	-150.31508140	**TS4-5**	-1678.58707260
**1**	-1291.68819170	**TS5-6**	-1183.13141220
**2**	-645.80150140	**TS6-7**	-1183.07489710
**3**	-386.98516990	**TS7-9**	-537.23753740
**3***	-386.90128300	**TS6-10**	-1183.10271770
**4***	-1678.60369590	**TS10-11**	-1183.15488520
**5**	-1032.81960430		
**6**	-1183.16427170		
**7**	-537.31757830		
**8**	-114.49265910		
**9**	-422.92041250		
**10**	-1183.17471160		
**11**	-760.23743540		
**12**	-760.28495310		
**13**	-1829.03210100		
**14**	-721.00909740		
**15**	-1108.00412460		
**16**	-685.07870210		
**17**	-1406.17097390		
**17***	-1406.09367870		
**18**	-2366.35496210		
**19**	-835.43942000		
**20**	-1520.62472690		
**20***	-1520.49208410		

***Triplet**

4. Cartesian Coordinates for Optimized Structures

Oxygen

E= -150.3717204 a.u., number of negative frequencies = 0

0 3

O	0.00000000	0.00000000	0.60266900
O	0.00000000	0.00000000	-0.60266900

1

E= -1291.9634216 a.u., number of negative frequencies = 0

0 1

C	3.87617300	1.48613100	0.43380900
C	3.91829100	0.65574200	-0.68155400
N	2.96364700	-0.23452500	-0.96599300
C	1.93164000	-0.32465500	-0.13481600
C	1.78207400	0.45471100	1.01273600
C	2.78360100	1.37736900	1.29164400
S	0.77340600	-1.59320100	-0.69835100
S	-0.77340600	-1.59320300	0.69834200
C	-1.93163900	-0.32465300	0.13481400
C	-1.78206800	0.45472700	-1.01272700
C	-2.78359600	1.37738600	-1.29163000
C	-3.87617300	1.48613600	-0.43380000
C	-3.91829700	0.65573100	0.68155200
N	-2.96365300	-0.23453800	0.96598500
H	4.67394800	2.19371200	0.62211200
H	4.74817700	0.70110900	-1.38044000
H	0.92370200	0.33800200	1.65983600
H	2.71054000	2.00423300	2.17339900
H	-0.92369300	0.33802700	-1.65982500
H	-2.71053100	2.00426200	-2.17337600
H	-4.67394900	2.19371700	-0.62209900

H	-4.74818700	0.70108800	1.38043300

2

E= -645.94569950 a.u., number of negative frequencies = 0

0 2

C	0.16856800	1.20841600	0.00000000
C	1.55735300	1.18606400	-0.00000100
C	2.20024800	-0.04609200	-0.00000100
C	1.41896600	-1.21526100	-0.00000300
N	0.09406000	-1.21807800	0.00000100
C	-0.54169100	-0.01604600	0.00001300
S	-2.27563800	-0.00768400	-0.00000300
H	-0.37519500	2.14398500	-0.00000400
H	2.12274000	2.11035000	-0.00000400
H	3.28081600	-0.11965300	-0.00000300
H	1.90277200	-2.18767400	-0.00000300

3

E= -387.17816720 a.u., number of negative frequencies = 0

0 1

C	2.28912300	1.39439200	-0.00007100
C	3.25666700	0.38586000	-0.00014800
C	2.85472500	-0.94887700	-0.00015900
C	1.49985700	-1.27070300	-0.00009600
C	0.51243300	-0.26870500	-0.00002000
C	0.93672500	1.07456500	-0.00000800
C	-0.89704700	-0.66831000	0.00004100
C	-1.97620400	0.14028800	0.00009500
C	-3.34043200	-0.35273900	0.00014500
C	-4.42610700	0.43457200	0.00019100
H	2.59312300	2.43563500	-0.00006000
H	4.31069000	0.64045400	-0.00019700

H	3.59589000	-1.74082900	-0.00021800
H	1.19490100	-2.31228000	-0.00010600
H	0.20542100	1.87458900	0.00005100
H	-1.06994100	-1.74321700	0.00003600
H	-1.85387800	1.22103900	0.00010100
H	-3.46632400	-1.43437400	0.00014200
H	-5.42785600	0.01914600	0.00022600
H	-4.34046700	1.51777100	0.00019500

3*

E= -387.10270410 a.u., number of negative frequencies = 0

0 3

C	2.23417000	1.42286200	-0.00008200
C	3.24131400	0.44535500	-0.00015300
C	2.88132500	-0.91618700	-0.00015600
C	1.55604200	-1.29098300	-0.00008900
C	0.49940500	-0.31375500	-0.00001600
C	0.89866500	1.06865300	-0.00001600
C	-0.83197700	-0.73274800	0.00004700
C	-2.01250800	0.13311700	0.00011700
C	-3.29559300	-0.35196700	0.00012900
C	-4.45816100	0.44635500	0.00019200
H	2.50718900	2.47302000	-0.00008000
H	4.28559100	0.73510500	-0.00020600
H	3.65541500	-1.67640500	-0.00021100
H	1.28715000	-2.34237300	-0.00009100
H	0.14430900	1.84532300	0.00003800
H	-1.02077200	-1.80322400	0.00003800
H	-1.86913200	1.20955700	0.00015900
H	-3.43109700	-1.43269700	0.00008700
H	-5.44396900	-0.00185900	0.00019900
H	-4.39077600	1.52934600	0.00023500

4

E= -1679.07930490 a.u., number of negative frequencies = 0

0 3

C	4.37938200	0.28900700	-0.92601200
C	4.63920400	1.66357500	-1.03990500
C	3.91691800	2.57056900	-0.24129300
C	2.96103700	2.11926500	0.64240700
C	2.66536100	0.71836900	0.77613200
C	3.42360800	-0.18362500	-0.04711200
C	1.67466800	0.30108300	1.66733100
C	1.21714900	-1.07172800	1.87497700
C	0.18299200	-1.39020800	2.71518300
C	-0.31191300	-2.69543400	2.92453300
S	-2.48820800	-1.62913700	0.28231200
S	-3.40112400	-0.10821500	-0.81619900
C	-0.97960400	-1.99844100	-0.64714200
C	-2.61022900	1.42633800	-0.28752800
N	-0.40266600	-1.02493700	-1.33828200
C	0.74366000	-1.30612900	-1.97160100
C	1.35634900	-2.55368400	-1.91838500
C	0.73677500	-3.56381600	-1.18417500
C	-0.46306300	-3.29200100	-0.53665200
C	-1.46486800	1.50858900	0.50364800
C	-0.97821300	2.77892800	0.78864600
C	-1.63534500	3.89772700	0.27730800
C	-2.76477600	3.69577400	-0.50849000
N	-3.25130100	2.47872300	-0.78717600
H	4.93523300	-0.41418200	-1.53755000
H	5.38910800	2.02360300	-1.73457400
H	4.11399700	3.63425400	-0.32375500
H	2.40603400	2.82489600	1.25171600
H	3.24160700	-1.24809400	0.01618600
H	1.17096400	1.06784800	2.24920300

H	1.69713300	-1.87194800	1.32088000
H	-0.30757400	-0.57851300	3.24975200
H	0.13278600	-3.54657900	2.42166600
H	-1.14055300	-2.87693100	3.59726200
H	1.19192200	-0.49060800	-2.52950400
H	2.29040000	-2.72518900	-2.43864700
H	1.17399400	-4.55389200	-1.12399200
H	-0.98191400	-4.05738300	0.02639000
H	-0.96713500	0.62167600	0.86573500
H	-0.08677100	2.89065500	1.39434900
H	-1.28053700	4.90117700	0.47717500
H	-3.30615300	4.53539100	-0.93311500

5

E= -1033.15081280 a.u., number of negative frequencies = 0

0 2

C	5.69244500	-0.20122700	0.27592800
C	5.91069800	1.17818200	0.19397400
C	4.83032900	2.03001500	-0.05102200
C	3.55279600	1.51117000	-0.21098100
C	3.30766100	0.11794300	-0.13132200
C	4.41820200	-0.72690100	0.11706200
C	1.96184600	-0.36195600	-0.30401000
C	1.54061900	-1.69861800	-0.24419600
C	0.25052600	-2.14664600	-0.41567600
C	-0.93119000	-1.28791600	-0.70926500
S	-1.74028800	-0.81091300	0.89810300
C	-3.24046600	-0.01432500	0.33105900
N	-3.43641600	0.13112500	-0.97787100
C	-4.56573800	0.73598300	-1.38011900
C	-5.53013200	1.21754300	-0.50615700
C	-5.31167600	1.05961600	0.86531500
C	-4.15380300	0.43387000	1.29943300

H	6.52609400	-0.86899100	0.46538300
H	6.90912500	1.58179800	0.31914200
H	4.98809000	3.10117000	-0.11679400
H	2.71898500	2.17945700	-0.40072200
H	4.27917100	-1.79913500	0.18462700
H	1.21511200	0.40385800	-0.49064800
H	2.28771900	-2.46106800	-0.04290900
H	0.05538800	-3.20788400	-0.30130800
H	-0.68239700	-0.36942900	-1.23865000
H	-1.68071700	-1.82943200	-1.28555800
H	-4.69219700	0.83486500	-2.45417700
H	-6.42280200	1.69973800	-0.88481300
H	-6.03777400	1.41885000	1.58594000
H	-3.95698200	0.29253700	2.35580700

6

E= -1183.54344020 a.u., number of negative frequencies = 0

0 2

C	4.86964700	-1.44747200	1.43526900
C	5.85274300	-1.65668200	0.46432500
C	5.67335600	-1.14306700	-0.81871400
C	4.52089300	-0.42404600	-1.12695900
C	3.52695300	-0.19985400	-0.15987800
C	3.71987500	-0.72941100	1.12898800
C	2.33945900	0.57563500	-0.53974700
C	1.33689000	0.96586100	0.25810700
C	0.16431400	1.73012200	-0.25156600
C	-1.19186200	1.18171000	0.17810600
O	0.27248000	3.10198100	0.35487400
O	-0.54061300	3.96239900	-0.21515600
S	-1.45327200	-0.46208800	-0.59552000
C	-3.17482000	-0.75320800	-0.19038600
N	-3.82889300	0.14699200	0.53771200

C	-5.11643900	-0.09893800	0.82938200
C	-5.79067500	-1.23852200	0.41396400
C	-5.08976700	-2.17629500	-0.34909000
C	-3.76054000	-1.93832700	-0.66177600
H	5.00087900	-1.84920700	2.43408000
H	6.74778400	-2.21827700	0.70798100
H	6.42904200	-1.30213400	-1.58006800
H	4.38552600	-0.02645100	-2.12766900
H	2.96608600	-0.58590300	1.89415400
H	2.28812800	0.85009700	-1.59155100
H	1.32403500	0.73365300	1.31913100
H	0.21192500	1.88538400	-1.33052900
H	-1.23627300	1.08186900	1.26358600
H	-1.98288700	1.85135100	-0.15472100
H	-5.61921300	0.65791900	1.42385700
H	-6.83009900	-1.38835400	0.67828300
H	-5.57564900	-3.08100300	-0.69666000
H	-3.18978500	-2.64456700	-1.25341500

7

E= -537.55567340 a.u., number of negative frequencies = 0

0 1

C	3.12873900	1.41826400	-0.04358600
C	4.11225800	0.42551300	-0.04782500
C	3.73581700	-0.91559700	-0.00394200
C	2.38698900	-1.26020200	0.04208100
C	1.38609200	-0.27411600	0.04244200
C	1.78267200	1.07535800	0.00218300
C	-0.01974300	-0.69711400	0.08241100
C	-1.10624000	0.08520200	0.01437600
C	-2.48503200	-0.45000400	0.08618500
C	-3.51727400	0.25314800	0.96210600
O	-4.41728100	0.33557000	-0.17870800

O	-3.30361200	-0.05652500	-1.08362100
H	3.41496600	2.46395800	-0.07364200
H	5.16124700	0.69798600	-0.08247800
H	4.49109800	-1.69389500	-0.00486000
H	2.09933400	-2.30605400	0.07611400
H	1.03651000	1.86144800	0.01148900
H	-0.17287900	-1.77086000	0.17703000
H	-1.02876600	1.16322300	-0.09724900
H	-2.49740500	-1.53836200	0.19159800
H	-3.95013300	-0.32094200	1.78268900
H	-3.21248700	1.24842300	1.29935200

8

E= -114.54095720 a.u., number of negative frequencies = 0

0 1

C	-0.52913700	0.00000000	-0.00000100
O	0.67677300	0.00000100	0.00000000
H	-1.11967700	-0.93754200	0.00000200
H	-1.11968800	0.93753700	0.00000200

9

E= -423.11257480 a.u., number of negative frequencies = 0

0 1

C	-2.29939200	1.37081200	0.00007600
C	-3.23505000	0.33214900	0.00013600
C	-2.80002400	-0.99223600	0.00013800
C	-1.43752200	-1.27636200	0.00008100
C	-0.48411300	-0.24280400	0.00002100
C	-0.93959600	1.08956900	0.00001900
C	0.93278000	-0.59655200	-0.00003400
C	1.99121600	0.23900500	-0.00009300
C	3.35011700	-0.28978900	-0.00013800

O	4.35696400	0.39831600	-0.00018600
H	-2.63507000	2.40182500	0.00007500
H	-4.29578300	0.55711000	0.00018100
H	-3.52053800	-1.80246800	0.00018500
H	-1.10076700	-2.30773400	0.00008400
H	-0.22719100	1.90597800	-0.00002600
H	1.14078900	-1.66584600	-0.00002400
H	1.89086900	1.31993800	-0.00010900
H	3.42147000	-1.39807600	-0.00011500

10

E= -1183.55729340 a.u., number of negative frequencies = 0

0 2

C	-3.38689700	-1.82946600	1.54987100
C	-4.42278600	-0.96551500	1.89418100
C	-4.66322600	0.15221500	1.09473900
N	-3.93883700	0.43362600	0.00502400
C	-2.95404400	-0.40488100	-0.29222800
C	-2.62168700	-1.54897400	0.41855400
S	-2.01283000	0.02442600	-1.80615700
C	-1.26808800	1.58461600	-1.14005500
O	-0.93728400	-1.04108800	-1.96752900
C	-0.42469200	1.47177100	0.16143800
C	0.82015500	0.62999700	0.00141600
O	-0.26400000	2.70310100	0.70387700
C	2.02564600	1.00909000	0.43829700
C	3.28074700	0.25448800	0.31831200
C	4.44510800	0.78325100	0.90062700
C	5.66098600	0.10756500	0.82219500
C	5.73985500	-1.11330300	0.15475200
C	4.59169300	-1.65068700	-0.43392000
C	3.37817900	-0.97774900	-0.35486600
H	-3.17463200	-2.70668400	2.14998700

H	-5.03991500	-1.15011400	2.76479500
H	-5.46395500	0.84437900	1.33368700
H	-1.80223200	-2.17735300	0.09411900
H	-0.65982000	1.98737700	-1.95267700
H	-2.12556800	2.23709000	-0.96717500
H	-1.08052800	0.95388200	0.90673500
H	0.65461400	-0.31501800	-0.50543500
H	2.10623300	1.97093100	0.93879200
H	4.38996600	1.73395800	1.42121200
H	6.54547900	0.53554600	1.28145200
H	6.68439000	-1.64203900	0.09022200
H	4.64562500	-2.59847000	-0.95868900
H	2.50204700	-1.41029300	-0.82434100

11

E= -760.42746100 a.u., number of negative frequencies = 0

0 2

C	-2.32892600	1.03164000	-0.16995600
C	-2.79522100	-0.25170700	0.10218000
C	-1.87103600	-1.28699300	0.24215600
N	-0.54902600	-1.10365800	0.13622200
C	-0.13400100	0.12698200	-0.12124100
C	-0.95587100	1.23519100	-0.29327300
S	1.70483300	0.35827300	-0.29417800
C	2.03020500	0.35444100	1.42884500
O	2.28376100	-0.90942400	-0.88587800
H	-3.01892900	1.85828300	-0.29370200
H	-3.85458500	-0.45438600	0.20122100
H	-2.20142600	-2.30002200	0.44909200
H	-0.54303600	2.21120200	-0.51930700
H	2.26206600	-0.59466300	1.89928700
H	1.98077900	1.29089200	1.97145400

12

E= -760.47653210 a.u., number of negative frequencies = 0

0 2

C	2.66677600	-0.69483400	-0.08961200
C	2.81471700	0.69342800	-0.04192800
C	1.66774300	1.47187100	0.03190400
N	0.43615900	0.94474400	0.05376600
C	0.29941600	-0.37815100	0.01410400
C	1.39529100	-1.24898200	-0.05779300
S	-1.35903300	-1.05857200	0.05776700
C	-2.33498000	0.53946700	0.34752200
O	-3.31819000	0.75998600	-0.48896700
H	3.53625800	-1.33970600	-0.14721800
H	3.79177500	1.15923500	-0.06401600
H	1.72486900	2.55489600	0.06882900
H	1.25114400	-2.32236000	-0.08416000
H	-2.60731700	0.59149900	1.41483000
H	-1.51357400	1.30371800	0.19765200

13

E= -1829.54679370 a.u., number of negative frequencies = 0

0 1

C	1.74410800	-0.48313000	-1.27656800
C	0.27681400	-0.77729200	-1.33112000
C	2.31255000	0.11037600	-0.22069700
C	3.72930100	0.45906100	-0.04545800
C	-0.39276900	-0.13839200	-2.55101500
O	0.03294700	-2.19407600	-1.48289400
O	0.34721300	-2.84650100	-0.18551500
S	-1.05231500	-3.19864100	0.67005000
C	-1.38709500	-1.66077600	1.54807100
S	-2.22261600	-0.16815400	-2.50262400

C	-2.57234600	1.13159500	-1.32068100
C	-3.91826800	1.40900700	-1.03402600
C	-4.19815800	2.43001500	-0.13907700
C	-3.14339800	3.13384700	0.44721800
C	-1.84808700	2.77124900	0.10591600
N	-1.55929700	1.79007200	-0.76285300
C	-2.69028800	-1.16244900	1.52394600
C	-2.95587200	-0.00075700	2.24433100
C	-1.91157300	0.62623300	2.91500800
C	-0.64036600	0.05512400	2.85150800
N	-0.37699200	-1.08114000	2.19896600
C	4.15154400	0.95775900	1.19825500
C	5.48425900	1.29526000	1.42372500
C	6.42484100	1.14389400	0.40633800
C	6.01953600	0.65444200	-0.83821700
C	4.68970100	0.31756200	-1.06356600
H	2.32129400	-0.78988700	-2.14522100
H	-0.21443300	-0.42141200	-0.42751900
H	1.67163400	0.35928000	0.62238400
H	-0.07171200	0.89891300	-2.63441900
H	-0.11247100	-0.66597400	-3.46545700
H	-4.71475500	0.84399600	-1.50400700
H	-5.22685900	2.67319700	0.10176700
H	-3.32120900	3.93430300	1.15443600
H	-0.99513000	3.27986700	0.54454800
H	-3.46260000	-1.65208500	0.94430500
H	-3.95304500	0.42148300	2.25024400
H	-2.06867100	1.54357000	3.46845800
H	0.20023500	0.52461000	3.35323000
H	3.42346400	1.07643400	1.99449500
H	5.78710800	1.67600700	2.39312300
H	7.46299300	1.40645900	0.57726300
H	6.74471300	0.53860600	-1.63654100

H	4.39448400	-0.05232200	-2.03875700

14

E= -721.17773320 a.u., number of negative frequencies = 0

0 2

C	1.73138000	1.30990600	0.00000500
C	2.54793800	0.17932500	-0.00000600
C	1.95296200	-1.08282500	-0.00000800
N	0.62784600	-1.26075800	-0.00000300
C	-0.12739100	-0.16314500	0.00000400
C	0.35048500	1.14513800	0.00001100
S	-1.88354200	-0.53217800	0.00001200
O	-2.62551200	0.80484100	-0.00002600
H	2.16357800	2.30381300	0.00000800
H	3.62719800	0.26838200	-0.00001200
H	2.56048200	-1.98202900	-0.00001400
H	-0.33765400	1.98086600	0.00002100

15

E= -1108.35807200 a.u., number of negative frequencies = 0

0 2

C	1.07112800	1.12930300	-0.14513600
C	-0.28829300	1.50333800	-0.69481000
C	1.84247400	0.19093400	-0.73244300
C	3.17936200	-0.23035300	-0.32375200
C	-1.27300800	1.98110500	0.38048200
O	0.14255100	2.54241100	-1.48906200
S	-1.80311100	0.65788200	1.52717400
C	-3.03914500	-0.21234100	0.56102000
C	-3.64606200	-1.33549900	1.14385800
C	-4.61256300	-2.00839900	0.41234100
C	-4.94344300	-1.55126500	-0.86586100

C	-4.28140700	-0.43142800	-1.35008100
N	-3.34426300	0.23206400	-0.65482400
C	3.79137600	-1.29185900	-1.01609900
C	5.06374200	-1.73469100	-0.66846500
C	5.75130900	-1.12628400	0.38107600
C	5.15714500	-0.07079800	1.07943400
C	3.88801400	0.37377900	0.73408900
H	1.42326800	1.73745500	0.68065100
H	-0.72305600	0.67200200	-1.26646200
H	1.42927900	-0.33647300	-1.58936100
H	-0.80616700	2.74545400	1.00513900
H	-2.16073600	2.40181400	-0.09091600
H	-3.36651400	-1.66545400	2.13763600
H	-5.10232300	-2.87971000	0.83230800
H	-5.69154100	-2.05056300	-1.46896000
H	-4.50250800	-0.04019600	-2.33857100
H	3.25855800	-1.76709500	-1.83312400
H	5.51861700	-2.55289300	-1.21555600
H	6.74320300	-1.46855400	0.65438800
H	5.68961900	0.40620500	1.89478700
H	3.44574000	1.19491100	1.28559700

16

E= -685.24366450 a.u., number of negative frequencies = 0

0 2

C	2.53612200	0.37652500	-0.00000100
C	2.14370100	-0.96453800	0.00000000
C	0.79188300	-1.27404600	0.00000100
C	-0.12224600	-0.21134400	0.00000100
N	0.23394800	1.06802400	0.00000100
C	1.54563800	1.35030200	-0.00000100
S	-1.86786500	-0.63322900	-0.00000100

C	-2.66726400	0.88795200	0.00000100
H	3.58155100	0.65870800	-0.00000100
H	2.88243500	-1.75788700	0.00000100
H	0.45205600	-2.30317700	0.00000100
H	1.80396500	2.40478400	0.00000000
H	-3.74789500	0.86289100	-0.00000100
H	-2.09089900	1.80107700	0.00000000

17

E= -1406.49113010 a.u., number of negative frequencies = 0

0 1

C	-4.85872700	1.22703900	0.35210700
C	-4.20051600	2.10725100	-0.50997800
C	-2.93950100	1.75437700	-0.97259500
N	-2.32195600	0.61101300	-0.63440000
C	-2.96682300	-0.21206100	0.18094000
C	-4.23776300	0.03954400	0.71191700
S	-2.15661700	-1.74754400	0.65891900
O	-0.77326000	-1.72299900	-0.29281800
C	0.30161600	-0.94459400	0.25449200
S	1.54734500	-0.97301100	-1.09558700
C	2.88350400	-0.09883500	-0.28973400
C	4.09354100	0.04222900	-0.98545600
C	5.12376800	0.73063900	-0.36398600
C	4.92390400	1.24922400	0.91811400
C	3.68873300	1.05066300	1.51891100
N	2.67752000	0.39073300	0.93174100
H	-5.84457700	1.46221900	0.73666300
H	-4.65362900	3.04252400	-0.81418400
H	-2.39017500	2.40889500	-1.64217900
H	-4.72258900	-0.66801700	1.37560300
H	-0.00874300	0.08038200	0.44897600
H	0.71178800	-1.40828800	1.15283900

H	4.21726500	-0.37585800	-1.97760500
H	6.07307200	0.86041500	-0.87124900
H	5.70517500	1.79146600	1.43600500
H	3.48747700	1.43407600	2.51459200

17*

E= -1406.43092960 a.u., number of negative frequencies = 0

0 3

C	-1.79685600	1.63552000	-1.73510000
C	-1.03709300	2.31199500	-0.78913600
C	-1.22659000	2.00658700	0.56870400
N	-2.08689000	1.09877300	1.00932200
C	-2.82776200	0.42788000	0.08920700
C	-2.70631300	0.67970900	-1.29679800
S	-3.92067700	-0.79481200	0.65555300
O	-0.96743200	-2.45502300	0.57443600
C	-0.24952300	-1.60502300	-0.11258900
S	1.50863700	-2.16613400	-0.53100100
C	2.38278900	-0.66723900	-0.08061500
C	3.73611800	-0.53251300	-0.41894600
C	4.38660100	0.63563400	-0.04838100
C	3.68033800	1.63197400	0.62991900
C	2.33987100	1.40772500	0.91263800
N	1.70826600	0.27739800	0.56997400
H	-1.68275700	1.84081300	-2.79296300
H	-0.30861600	3.05941100	-1.07776500
H	-0.64387000	2.52316900	1.32513100
H	-3.31414200	0.12407800	-1.99884400
H	-0.72212200	-1.23357200	-1.03664300
H	-0.02417700	-0.68368000	0.50409300
H	4.25445400	-1.31682300	-0.95704700
H	5.43441100	0.77135600	-0.29097400
H	4.15647900	2.55601900	0.93266000

H	1.74332500	2.14786100	1.43554400

18

E= -2367.11074470 a.u., number of negative frequencies = 0

0 1

C	-7.34346100	1.17552700	0.33354400
C	-7.17764200	2.52428100	0.65962100
C	-5.89474000	3.02706200	0.86916800
C	-4.78875400	2.18809700	0.75210400
C	-4.93858800	0.83087500	0.42108000
C	-6.24010400	0.33784900	0.21709700
C	-3.73251000	0.00035500	0.29958200
C	-3.65589900	-1.27215300	-0.10665800
C	-2.36181700	-2.02814700	-0.15507000
C	-2.25271100	-3.02374000	1.00405000
S	-0.60079700	-3.79119800	1.18308800
C	0.37830500	-2.39557300	1.73026600
N	-0.24013200	-1.26952700	2.07843100
C	0.51328900	-0.24018900	2.49706800
C	1.89589900	-0.29442100	2.59261700
C	2.53430100	-1.48096600	2.22215000
C	1.77275600	-2.54981000	1.77761100
O	-2.29688100	-2.82860600	-1.37202800
O	-1.72507500	-2.03760600	-2.42452200
O	-0.40297500	-2.48290400	-2.62152200
O	0.46792900	-1.85099500	-1.65551900
C	1.18727100	-0.75880300	-2.29374900
C	0.26945000	0.38660700	-2.73415200
C	2.20969300	-0.36727300	-1.27399600
C	3.52675700	-0.46947200	-1.48007900
C	4.57209900	-0.01936100	-0.55067600
C	5.86977000	-0.54396800	-0.66468600
C	6.87820500	-0.15608800	0.21455800

C	6.61265500	0.77738500	1.21633800
C	5.33193700	1.32338200	1.32765500
C	4.32322900	0.93181400	0.45432800
S	-0.67261000	1.21882400	-1.40107000
C	0.51143800	2.40586800	-0.75976000
C	0.28282500	2.91593100	0.52635200
C	1.18118300	3.84619900	1.03094200
C	2.27530800	4.22810500	0.25326900
C	2.41206600	3.65868900	-1.00711400
N	1.54732200	2.76784600	-1.51252400
H	-8.33911500	0.77612700	0.17316000
H	-8.04139600	3.17352500	0.75060400
H	-5.75386200	4.07196300	1.12357200
H	-3.79163900	2.58524600	0.91379100
H	-6.39129500	-0.70670600	-0.02985900
H	-2.80599400	0.50168300	0.56691700
H	-4.53698400	-1.83818800	-0.39756200
H	-1.51054100	-1.35256200	-0.14175300
H	-2.49795400	-2.51210600	1.93319200
H	-2.94924600	-3.85449300	0.86926800
H	-0.02609300	0.66416000	2.76057900
H	2.45773000	0.56656300	2.93047600
H	3.61413000	-1.56119500	2.26205800
H	2.23933100	-3.47619800	1.46434200
H	1.67426700	-1.15404900	-3.19126300
H	0.86588200	1.14030400	-3.24792600
H	-0.48429900	0.01462800	-3.42784400
H	1.82060800	0.02504000	-0.34154100
H	3.87972000	-0.92011200	-2.40551400
H	6.08246400	-1.26820200	-1.44447900
H	7.87193200	-0.57898800	0.11483000
H	7.39830300	1.08553600	1.89730600
H	5.12087700	2.06272200	2.09272500

H	3.34082100	1.37712100	0.54205100
H	-0.56494200	2.58316100	1.11268600
H	1.03687400	4.25718300	2.02378000
H	3.00576900	4.94090700	0.61507900
H	3.25156700	3.91920800	-1.64434200

19

E= -835.65471280 a.u., number of negative frequencies = 0

0 2

C	-3.25785800	0.81331700	-0.00000200
C	-2.07400600	1.53777000	0.00001100
N	-0.85921100	0.96580400	0.00000800
C	-0.80231800	-0.36295100	-0.00000700
C	-1.93289100	-1.19074200	-0.00001300
C	-3.17859200	-0.58140600	-0.00001400
S	0.81184300	-1.14193300	-0.00001900
C	1.84351000	0.37149800	0.00005000
O	3.20776700	-0.14644200	0.00012400
O	4.07559500	0.84221400	-0.00012100
H	-4.21253300	1.32421500	0.00000000
H	-2.08570600	2.62323900	0.00002200
H	-1.83632800	-2.26991900	-0.00001600
H	-4.07791500	-1.18659400	-0.00002300
H	1.69181000	0.96414700	-0.90016300
H	1.69170600	0.96412700	0.90026000

20

E= -1520.99394080 a.u., number of negative frequencies = 0

0 1

C	-6.19062800	-1.10753300	0.74568300
C	-4.98733700	-1.62353400	0.28146100
N	-3.97544600	-0.85650700	-0.15060100

C	-4.14058400	0.45933500	-0.12655600
C	-5.30828600	1.08612600	0.32530700
C	-6.34893700	0.27915400	0.76400700
S	-2.81182300	1.52256000	-0.72616400
C	-1.47602300	0.33776000	-0.99201300
O	-0.84684400	0.12389000	0.27727000
O	0.22944600	-0.83535000	0.03029700
C	1.43321100	-0.07667400	0.06420100
S	2.70493000	-1.30471000	-0.44665300
C	4.18582700	-0.38541700	-0.03232400
N	4.06368200	0.89295800	0.31659800
C	5.18498200	1.57055900	0.60985200
C	6.45326500	1.00933200	0.55490200
C	6.56454000	-0.33249100	0.18240600
C	5.41678200	-1.05210600	-0.11354800
H	-6.97698500	-1.77053300	1.08437300
H	-4.81650700	-2.69523400	0.25176500
H	-5.39629500	2.16629100	0.33322900
H	-7.26959100	0.72681000	1.12053000
H	-1.88107700	-0.59638900	-1.37984000
H	-0.78208000	0.79619800	-1.70141600
H	1.40253200	0.75268100	-0.64437900
H	1.65325700	0.28341800	1.07011700
H	5.04862200	2.60880400	0.89648500
H	7.32639400	1.60243900	0.79678800
H	7.53467500	-0.81334800	0.12964700
H	5.46801200	-2.09727900	-0.39500800

20*

E= -1520.86981920 a.u., number of negative frequencies = 0

0 3

C	-5.07639300	-2.26535500	-0.56537900

C	-3.90530200	-1.82446500	-1.16879800
N	-3.30409300	-0.66833100	-0.85154900
C	-3.86068600	0.08236300	0.08992400
C	-5.03524100	-0.26769500	0.76688400
C	-5.65002500	-1.46371000	0.42272300
S	-3.09569200	1.65859100	0.52164300
C	-1.53154000	1.56998700	-0.37644800
O	-0.63015800	0.79946500	0.42598300
O	0.61665800	0.72074700	-0.34550500
C	1.51955700	1.61504100	0.30110800
S	3.06622600	1.56724300	-0.65032100
C	3.88023600	0.16122100	0.02152700
N	3.39504900	-0.49945200	1.03059500
C	3.99471900	-1.66411000	1.37599900
C	5.01398600	-2.30479100	0.48535700
C	5.54873300	-1.58479600	-0.53560900
C	5.11221800	-0.24236200	-0.73990400
H	-5.52257200	-3.20764800	-0.85808300
H	-3.42041600	-2.41565700	-1.93949300
H	-5.44999700	0.37316800	1.53615100
H	-6.56163800	-1.76900500	0.92367300
H	-1.68697700	1.09134600	-1.34276800
H	-1.17626700	2.59730600	-0.49430500
H	1.15957100	2.64666800	0.26476900
H	1.72471500	1.29777300	1.32400300
H	3.67536400	-2.14390700	2.29163400
H	5.30535500	-3.33022600	0.67891700
H	6.30143900	-2.01266700	-1.18949700
H	5.53261800	0.40432000	-1.49760900

TS4-5

E= -1679.07637240 a.u., im. freq.= 415.96*i*

0 1

C	2.61875000	-1.24668800	0.50590800
C	1.58798500	-1.17718600	-0.40108700
C	0.23421800	-1.20926200	-0.03254900
C	-0.81098600	-1.10250000	-0.97521300
S	-1.62216400	0.95342000	-1.01911100
S	-2.69944900	0.24907400	1.38040000
C	4.03398900	-1.20953000	0.21971300
C	4.94734900	-1.26408500	1.29701900
C	6.31859400	-1.22312300	1.07949900
C	6.81928700	-1.12769800	-0.22019900
C	5.93188600	-1.07428100	-1.30053300
C	4.56175200	-1.11496800	-1.08948500
C	-0.24442500	1.86176000	-0.40440200
C	-4.12501700	-0.45961800	0.62862300
C	-5.33704700	0.25637000	0.57533900
C	-6.44463400	-0.32861400	-0.02402000
C	-6.31793600	-1.60107000	-0.57815400
C	-5.07517200	-2.22948100	-0.50430800
N	-4.00433900	-1.69088200	0.08410000
C	0.31444700	2.87675000	-1.20293800
C	1.43360700	3.53940000	-0.72482600
C	1.96853700	3.17283800	0.51446400
C	1.33614600	2.16606600	1.22977900
N	0.24263000	1.52272600	0.79332000
H	2.35235000	-1.32952600	1.55712200
H	1.80931400	-1.08119500	-1.46096400
H	-0.02290100	-1.28059400	1.01727700
H	-0.53762400	-1.17059600	-2.02328400
H	-1.76797500	-1.55326100	-0.72423100
H	4.56262900	-1.33724400	2.30892500
H	6.99951600	-1.26570400	1.92243200
H	7.88919400	-1.09596900	-0.39256300
H	6.31642700	-1.00132800	-2.31195700

H	3.89383700	-1.07352600	-1.94140300
H	-5.39370300	1.24938800	1.00217600
H	-7.38863700	0.20323700	-0.06588300
H	-7.15297000	-2.09578200	-1.05895000
H	-4.93560100	-3.21717200	-0.93505900
H	-0.10914000	3.12013500	-2.16957500
H	1.89404200	4.32232000	-1.31663400
H	2.84865300	3.66046700	0.91504400
H	1.71237500	1.85305700	2.19919700

TS5-6

E= -1183.52851890 a.u., im. freq.= 187.38*i*

0 2

C	5.55504300	-0.34323200	0.98407100
C	6.23675400	-0.91965200	-0.09226500
C	5.54617300	-1.21247300	-1.26880100
C	4.18819800	-0.93205900	-1.36764300
C	3.48317700	-0.35052800	-0.29269200
C	4.19923600	-0.06103700	0.88961100
C	2.06744300	-0.08154100	-0.45630400
C	1.21066500	0.46439000	0.46396100
C	-0.14259300	0.71526800	0.17532200
C	-1.12462800	1.09464200	1.21618900
O	0.01539200	2.71465800	-0.75074400
O	-0.94274000	3.47947200	-0.53325300
S	-2.09631300	-0.36267300	1.81668300
C	-3.27203400	-0.60517100	0.48472600
N	-3.25076200	0.22389100	-0.55450200
C	-4.14752600	0.02807900	-1.53394800
C	-5.08956700	-0.99103800	-1.51460700
C	-5.09917200	-1.85655200	-0.41763500
C	-4.17857700	-1.66990200	0.60206700

H	6.08780900	-0.11326000	1.90031900
H	7.29596200	-1.13706500	-0.01213700
H	6.06681800	-1.65950600	-2.10845500
H	3.65470500	-1.16128400	-2.28433100
H	3.69339900	0.38703700	1.73630300
H	1.65102800	-0.35170500	-1.42435800
H	1.57184000	0.75361700	1.44696200
H	-0.55245900	0.34697400	-0.75802600
H	-0.64232700	1.49041900	2.11163700
H	-1.84121000	1.82604200	0.83724900
H	-4.09843000	0.72419500	-2.36581100
H	-5.79141300	-1.10582300	-2.33125700
H	-5.81597300	-2.66790600	-0.35948700
H	-4.16016900	-2.32440800	1.46563900

TS6-7

E= -1183.46703130 a.u., im. freq.= 1602.96*i*

0 2

C	1.66635600	0.12018300	-0.55257500
C	1.09900900	1.24016900	-0.07812300
C	-0.30540100	1.60059900	-0.38668800
C	-1.15041500	2.17078700	0.74057400
S	-1.45438600	-0.18080500	1.68830400
C	-2.73413200	-0.76008900	0.61714100
N	-3.23118000	0.08254000	-0.30892600
C	-4.19682000	-0.35918000	-1.12063100
C	-4.72431900	-1.64709700	-1.06816200
C	-4.21220100	-2.52320900	-0.11021600
C	-3.21039700	-2.08261300	0.74141200
O	-0.29709200	2.87308000	-1.16445400
O	-0.87480100	3.67112700	-0.20890000
C	3.03515000	-0.34664800	-0.32362100

C	3.42118200	-1.58462100	-0.86633100
C	4.70940400	-2.07834600	-0.68005500
C	5.63869600	-1.34213000	0.05314300
C	5.27020100	-0.10879800	0.59816100
C	3.98510400	0.38515100	0.41372500
H	1.05199500	-0.53740100	-1.16377800
H	1.64102400	1.94959200	0.53990500
H	-0.82383300	0.85289600	-0.98523600
H	-0.70951900	2.43951200	1.68848000
H	-2.22651400	2.12564600	0.65979600
H	-4.56768300	0.35615200	-1.84963700
H	-5.50760300	-1.94802400	-1.75280700
H	-4.58962300	-3.53660800	-0.02873400
H	-2.79308800	-2.73857300	1.49522400
H	2.69963800	-2.15982200	-1.43730600
H	4.98717200	-3.03569700	-1.10680800
H	6.64323600	-1.72306000	0.19986600
H	5.99039800	0.46781600	1.16809600
H	3.71947600	1.34406100	0.84287200

TS7-9

E= -537.49800990 a.u., im. freq.= 1269.81*i*

0 1

C	-0.03726500	-0.53186100	-0.23947200
C	-1.08530300	0.32580400	-0.12253800
C	-2.47177500	-0.10481600	-0.27618800
C	-3.51174000	-0.37971000	0.98402700
O	-4.58463800	-0.19559100	0.19729000
O	-3.21023900	0.85002100	-0.92728400
C	1.37226300	-0.22486400	-0.09429800
C	2.30404700	-1.27912800	-0.18828100
C	3.66629500	-1.03942800	-0.05853400

C	4.12532800	0.25893100	0.16640800
C	3.21487900	1.31718900	0.26059200
C	1.85463600	1.08271900	0.13174100
H	-0.26801700	-1.57524300	-0.44715300
H	-0.93480900	1.37404400	0.11507300
H	-2.53234100	-1.10148400	-0.73473900
H	-3.36296900	-1.40241100	1.35150600
H	-3.36894500	0.38215900	1.76596100
H	1.94464800	-2.28757100	-0.36315200
H	4.37016200	-1.86040000	-0.13287400
H	5.18795600	0.44924300	0.26733500
H	3.57378500	2.32542200	0.43309900
H	1.16134100	1.91178400	0.20415200

TS6-10

E= -1183.49217820 a.u., im. freq.= 1007.13*i*

0 2

C	-5.34761300	1.89030700	0.25779700
C	-6.49327600	1.17489500	-0.10059600
C	-6.38049500	-0.16760800	-0.45707400
C	-5.13325100	-0.78810100	-0.45334100
C	-3.97210900	-0.08370300	-0.09289200
C	-4.10275200	1.27185400	0.26138800
C	-2.68579300	-0.79555700	-0.10298100
C	-1.48665800	-0.31192800	0.24447500
C	-0.22659000	-1.12729300	0.19422100
C	0.79877100	-0.48414300	-0.75200600
O	0.32309800	-1.14736100	1.50434700
O	1.78461200	-1.86985700	1.15240800
S	2.42099800	-1.34717900	-0.48732700
C	3.57196000	0.01236500	-0.30780900
N	3.11091400	1.22031300	0.01056900

C	4.00705500	2.19588100	0.19676100
C	5.38097300	2.00193900	0.08080600
C	5.84797900	0.72835700	-0.24959400
C	4.93267800	-0.29532600	-0.44881600
H	-5.42709900	2.93613000	0.53427200
H	-7.46202700	1.66207700	-0.10241100
H	-7.26249400	-0.73280800	-0.73803800
H	-5.05180300	-1.83409100	-0.73131900
H	-3.22797400	1.84841100	0.53888900
H	-2.73998500	-1.83092600	-0.43455500
H	-1.35953100	0.70785000	0.59639100
H	-0.44874300	-2.14698300	-0.14614700
H	0.53487500	-0.59051400	-1.80399100
H	0.97241000	0.56104900	-0.49690200
H	3.60443300	3.17144400	0.45037300
H	6.06324100	2.82667300	0.24428200
H	6.90950300	0.53667900	-0.35402300
H	5.25585800	-1.29621400	-0.70963900

TS10-11

E= -1183.54320260 a.u., im. freq.= 411.24*i*

0 2

C	3.19614600	-1.94589100	-1.40411500
C	4.35146400	-1.19383700	-1.59975400
C	4.60301600	-0.11030400	-0.75852100
N	3.77489100	0.24244400	0.23296100
C	2.67992500	-0.48610500	0.38121900
C	2.32289500	-1.59026700	-0.37691000
S	1.59534600	0.03476400	1.76857100
C	1.20552300	1.68063000	1.19035700
O	0.37791500	-0.86439700	1.77142500
C	0.47736100	1.88320700	-0.68181300
C	-0.61555000	0.88525500	-0.58327100
O	0.27811100	3.12542700	-0.71249000

C	-1.91258700	1.23243000	-0.51295000
C	-3.03286800	0.30823700	-0.34231100
C	-4.33213200	0.72231400	-0.68451700
C	-5.41553400	-0.14134900	-0.54931700
C	-5.22353900	-1.43144200	-0.05458800
C	-3.94115800	-1.84824400	0.31311000
C	-2.85658300	-0.99007700	0.17542100
H	2.97390400	-2.79527600	-2.03943600
H	5.05275500	-1.44034500	-2.38720500
H	5.49592900	0.49381000	-0.87930100
H	1.40851900	-2.13176600	-0.17262900
H	0.40517200	2.12143500	1.77940500
H	2.11064200	2.27900400	1.11666500
H	1.40867700	1.48152000	-1.12461800
H	-0.30998900	-0.15305000	-0.54912600
H	-2.15889700	2.28827000	-0.59981600
H	-4.48367100	1.72648300	-1.06716800
H	-6.40956900	0.19243900	-0.82628500
H	-6.06702100	-2.10393100	0.05612400
H	-3.79129600	-2.84252800	0.71986800
H	-1.87142000	-1.30499400	0.50193600
